# Dangers of Travel

**Published:** 1875-01

**Authors:** 


Dangers of Travel.—We are improving in the matter of safety in traveling. Taking travel by steam vessels in the United States, in 1873, we find that the number of lives lost from all causes were 222, while the average for the four years immediately preceding was 356. The number of lives lost by explosion is only 10, which is by far the smallest number ever recorded in one year, the total for the five years being 417, and the average for four corresponding years being about 102. The number of lives lost by fire in 1873 is 119, being greater than from all other causes combined. For 1872 the lives lost by fire bear even a larger proportion to the aggregate loss from other causes. The same is true of 1869. This is a matter demanding most serious attention and inquiry on the part of inspectors and owners of steam vessels, and there cannot be a doubt that thorough and earnest investigation into the causes which produce such disasters will be attended with the best results. Surely in every case either the construction, arrangement, equipment, or management is faulty in some particular, which a searching investigation must disclose. It is believed that a more thorough scrutiny on the part of inspectors in making their examinations, and like watchful care in the management of steamers by their officers, is only required to cause loss of life by fire to be an extremely rare occurrence, if it did not cease to have a place in our record. The lives lost by collisions in 1873 were 7. At one time collisions were a fruitful source of loss of life; but by adopting more perfect regulations, and by the experience gained in their use, loss from this cause seems to be almost uniformally reduced to about the number here stated. The lives lost by snags, wreck, and sinking are 86, which is about 10 per cent less than the average for the four preceding years. The lives lost in 1869 from all causes were 577; in 1870, 180; in 1871, 361; in 1872, 306; and in 1873, 222. These details show the year 1873 to have been a successful one in attaining in a high degree the aims of laws for the protection of life on steam vessels; but there is reason to believe that through improved administration and better regulation of service the results will be far surpassed in the future.

